# Physician preferences in diagnostics and treatment of juvenile osteochondritis dissecans are diverse across the knee, ankle and elbow: an ESSKA survey

**DOI:** 10.1007/s00167-023-07563-0

**Published:** 2023-10-03

**Authors:** Laura A. M. Kemmeren, Christiaan J. A. van Bergen, Max Reijman, Tom M. Piscaer

**Affiliations:** 1 Department of Orthopaedic Surgery and Sports Medicine, Erasmus Medical Centre, Sophia Children’s Hospital, Dr. Molewaterplein 40, 3015GD Rotterdam, The Netherlands; 2 Department of Orthopaedic Surgery, Amphia Hospital, Breda, The Netherlands

**Keywords:** Osteochondritis dissecans, Juvenile osteochondritis dissecans, Osteochondral defect, Elbow, Knee, Ankle, Adolescent, Paediatric, Cartilage, Survey, ESSKA, Arthroscopy, Drilling

## Abstract

**Purpose:**

To investigate the current preferences regarding the work-up and treatment choices of juvenile osteochondritis dissecans (JOCD) of the knee, ankle and elbow among orthopaedic surgeons.

**Methods:**

An international survey was set up for all European Society of Sports Traumatology, Knee Surgery and Arthroscopy (ESSKA) members, which assessed various questions on diagnosis and treatment of JOCD of different joints. Respondents answered questions for one or more joints, based on their expertise. Proportions of answers were calculated and compared between joints. Consensus was defined as more than 75% agreement on an item; disagreement was defined as less than 25% agreement.

**Results:**

Fifty physicians responded to the survey, of whom forty-two filled out the questions on the knee, fourteen on the ankle and nine on the elbow. Plain radiography and MRI were the most used imaging modalities for the assessment and follow-up of JOCD in the knee and ankle, but not for the elbow. MRI was also the preferred method to assess the stability of a lesion in the knee and ankle. There was universal agreement on activity and/or sports restriction as the non-operative treatment of choice for JOCD. Size, stability and physeal closure were the most important prognostic factors in determining the operative technique for the elbow. For the knee, these factors were size and stability and for the ankle, these were size and location.

**Conclusion:**

Activity and/or sports restriction was the non-operative treatment of choice. Furthermore, plain radiography and MRI were the preferred imaging modalities for the knee and ankle, but not for the elbow. For determining the operative technique, physicians agreed that the size of the lesion is an important prognostic factor in all joints. These findings help us understand how juvenile osteochondritis dissecans is treated in current practice and may provide opportunities for improvement.

**Level of evidence:**

Level V.

**Supplementary Information:**

The online version contains supplementary material available at 10.1007/s00167-023-07563-0.

## Introduction

Osteochondritis dissecans (OCD) is a disorder of the articular cartilage and subchondral bone that is frequently seen in children participating in sports [5, 18]. Juvenile osteochondritis dissecans (JOCD) occurs in skeletally immature children, i.e. children with open physes. The disorder may occur in different joints, the most common being the knee, ankle and elbow, respectively [21]. Irrespective of the location, JOCD may cause pain and mechanical symptoms in the affected joint, which could limit the mobility and athletic activities of the patient [3].

JOCD is a relatively rare condition, but it can greatly impact a child’s life [24]. So far, almost no randomised controlled trials have been performed and relatively little research has been conducted around the treatment of this condition. The aetiology of JOCD is not entirely clear [24]. This might be another reason this condition has no clear optimal treatment, resulting in a lack of standard treatment protocols. Additionally, there have only been a few consensus meetings [4].

JOCD in children appears to have better healing potential than OCD in adults [11, 14, 30]. Probably because of this, treatment choices differ between children and adults. Although certain features, such as lesion size and stability, must be considered for each JOCD, different joints may also require different treatments [21]. On the other hand, treatment principles between joints may have much in common.

An orthopaedic landscape gradually changing from general orthopaedic surgeons to joint specialists potentially causes us to work on ‘islands’, which may create knowledge segregation. Instead, we could learn from each other in this situation, as this same pathology affects different joints. Therefore, an international survey was developed to investigate current practices and preferences on the diagnosis and treatment of JOCD of the knee, ankle and elbow among orthopaedic surgeons.

## Materials and methods

An online survey was developed using Microsoft Forms. This survey included questions regarding symptoms, physical examination, imaging, non-operative and operative treatment and follow-up of JOCD. Similar questions were created for the knee, ankle and elbow. The respondents could answer the questions for all the joints for which they treated JOCD. There were 5 general questions and 21 questions per joint, including 2 cases per joint. Before they could submit the survey, all questions had to be answered for the joints physicians stated to treat. The survey is provided in the supplementary material.

### Reach

The survey was placed on the website of the European Society of Sports Traumatology, Knee Surgery and Arthroscopy (ESSKA). It was also available through the web link, which was provided at the ESSKA congress in Paris in 2022. The web link was also distributed through social media platform LinkedIN.

### Survey

#### General questions

The general questions included nationality, current profession, the number of years in practice and the approximate number of annual JOCD cases. Respondents were also asked to provide their definitions of JOCD.

#### Diagnosis

Respondents were asked how many JOCD lesions they annually treat per joint. Furthermore, respondents had to name differentiating symptoms and findings in the physical examination, which they thought were suggestive of JOCD. The use of imaging modalities, timing of imaging and imaging of the contralateral joint was also assessed. Furthermore, respondents were asked whether they determined skeletal age or requested genetic testing for JOCD.

#### Treatment

Physicians were asked what their preferred non-operative treatment plan was for JOCD and after what time or in what stage they would move on to operative treatment if symptoms were not alleviated. Respondents were also asked which types of operative treatment they used, which prognostic factors were most important in deciding what operative technique to use and which type of treatment they would use in two different cases (see supplementary material).

#### Follow-up

Physicians were also asked for what period of time they followed up on their patients, what imaging modalities they used for monitoring recovery and whether they recorded any patient-reported outcome measures (PROMs).

### Analysis

Absolute numbers and percentages are given for each question. Consensus was set at 75% agreement [27]. Likewise, disagreement on a statement was set at less than 25% of respondents opting for that item. Analysis was done using Microsoft Excel.

## Results

### General questions

Fifty physicians filled out the survey. Their answers to the general questions are given in Table 1.

**Table 1:** Respondent characteristics^a^

	*N* = 50
Profession	
Orthopaedic surgeon	41 (82)
Orthopaedic surgeon in training	9 (18)
Years of experience since residency	
0–5	6 (12)
5–10	9 (18)
10–15	9 (18)
15–20	10 (20)
20 +	8 (16)
Country of practice	
The Netherlands	21 (42)
Greece	4 (8)
Portugal	3 (6)
Germany	2 (4)
Spain	2 (4)
Other	11 (22)
Did not specify	7 (14)
Cases treated annually	
1–10	27 (54)
10–20	18 (36)
20 +	5 (10)
Key factor in definition of JOCD	
Skeletal maturity	23 (46)
Age	2 (4)
Both	26 (52)

^a^Numbers are given as absolute number (percentage)

### Knee

Forty-two physicians reported treating JOCD lesions of the knee (84%). Consensus was reached that during the patient interview, joint effusion and pain during activities are the most characteristic findings for JOCD (Table 2). For the physical examination, consensus was only obtained for joint effusion (88%).

**Table 2:** Outcomes of the survey per joint^a, b^

Joint	Knee (*n* = 42)	Ankle (*n* = 14)	Elbow (*n* = 9)
Lesions treated annually			
1–5	14 (33)	6 (43)	7 (78)
5–10	11 (26)	4 (29)	1 (11)
10–20	13 (31)	2 (14)	1 (11)
20 +	4 (10)	2 (14)	0 (0)
Characteristic findings during patient interview			
Pain on radial side of elbow	N/A	N/A	**9 (100)**
Pain on ulnar side of elbow	N/A	N/A	*1 (11)*
Participation in a throwing sport	N/A	N/A	5 (56)
Joint effusion	**37 (88)**	**11 (79)**	N/A
Pain during activities	**33 (79)**	**13 (93)**	*2 (22)*
Locking	28 (67)	8 (57)	6 (67)
Clicking	19 (45)	4 (29)	N/A
History of trauma	*7 (17)*	*3 (21)*	*1 (11)*
Limited range of motion	*7 (17)*	5 (36)	N/A
Pain at night	*1 (2)*	*1 (7)*	*1 (11)*
Instability	*1 (2)*	*2 (14)*	*0 (0.0)*
Characteristic findings during physical examination			
Joint effusion	**37 (88)**	9 (64)	6 (67)
Point tenderness on palpation	27 (64)	8 (57)	6 (67)
Crepitus	*10 (24)*	6 (43)	5 (56)
Limited range of motion	17 (40)	5 (36)	5 (56)
Positive radiocapitellar test	N/A	N/A	3 (33)
Positive Wilson’s test	*6 (14)*	N/A	N/A
Abnormal gait pattern	*10 (24)*	N/A	N/A
Limited stability/ligamentous laxity	*1 (2)*	4 (29)	N/A
Preferred imaging modality			
Plain radiography	**40 (95)**	**13 (93)**	5 (56)
CT	*8 (17)*	7 (50)	5 (56)
MRI	**42 (100)**	**12 (86)**	5 (56)
Radiography views			
AP	**37 (88)**	**12 (86)**	N/A
Lateral	**40 (95)**	**13 (93)**	5 (56)
AP in extension	N/A	N/A	5 (56)
AP in 45° flexion	N/A	N/A	*2 (22)*
Radial head	N/A	N/A	*2 (22)*
Rosenberg	11 (26)	N/A	N/A
Tunnel	20 (48)	N/A	N/A
Sunrise	*4 (10)*	N/A	N/A
Whole leg	*6 (14)*	N/A	N/A
Mortise	N/A	8 (57)	N/A
Heel rise	N/A	*1 (7)*	N/A
Horizontal beam lateral	N/A	*1 (7)*	N/A
Imaging of one or both joints			
One	22 (52)	5 (36)	6 (67)
Both	20 (48)	9 (64)	3 (33)
Preferred method of determining stability^c^			
History of patient	13 (31)	4 (29)	*1 (11)*
Physical examination	*6 (14)*	*2 (14)*	*1 (11)*
Range of motion	*1 (2)*	*3 (21)*	*1 (11)*
Plain radiography	*8 (19)*	*2 (14)*	*0 (0)*
CT	*3 (7)*	6 (43)	4 (44)
MRI	**41 (98)**	**12 (86)**	6 (67)
Arthroscopy	20 (48)	6 (43)	4 (44)
Preferred non-operative treatment			
Activity/sports restriction	**41 (98)**	**14 (100)**	**8 (89)**
Limited weight bearing	20 (48)	9 (64)	N/A
Immobilisation through casting	*0 (0)*	*1 (7)*	*1 (11)*
Immobilisation through bracing	*6 (14)*	*3 (21)*	*0 (0)*
Non-steroidal anti-inflammatory drugs	*9 (21)*	*1 (7)*	*1 (11)*
Injection therapy through corticosteroids	*1 (2)*	*0 (0)*	*0 (0)*
Injection therapy through platelet-rich plasma	*3 (7)*	*3 (21)*	*1 (11)*
Operative treatment arsenal			
Drilling	N/A	9 (64)	2 (22)
Internal fixation with non-absorbable devices	17 (40)	4 (29)	2 (22)
Internal fixation with bioabsorbable devices	26 (62)	7 (50)	3 (33)
Bone grafting	10 (24)	4 (29)	1 (11)
Debridement	22 (50)	10 (71)	7 (78)
Microfracturing	20 (48)	9 (64)	6 (67)
Autologous chondrocyte implantation	4 (10)	0 (0)	1 (11)
Osteochondral autograft plugs	14 (33)	3 (21)	2 (22)
Osteochondral allograft	3 (7)	2 (14)	0 (0)
Loose body removal	23 (55)	8 (57)	7 (78)
Antegrade drilling	20 (48)	N/A	N/A
Retrograde drilling	21 (50)	N/A	4 (44)
Transarticular drilling	3 (7)	N/A	N/A
Use of biologicals^d^			
No	36 (86)	10 (71)	8 (89)
Yes, platelet-rich plasma	6 (14)	3 (21)	1 (11)
Yes, bone marrow aspirate concentrate	2 (5)	2 (14)	0 (0)
Most prognostic factor for determining operative technique			
Size of lesion	**33 (79)**	**13 (93)**	**8 (89)**
Stability of lesion	**36 (86)**	8 (57)	**7 (78)**
Location of lesion	17 (40)	**11 (79)**	*2 (22)*
Lesion of cartilage only vs osteochondral lesion	17 (40)	4 (29)	*2 (22)*
Physeal closure	16 (38)	6 (43)	**8 (89)**
High-performing athlete	*4 (10)*	*1 (7)*	*0 (0)*
Imaging modality during follow-up after operative treatment			
Plain radiography	29 (69)	8 (57)	5 (56)
CT	*2 (5)*	5 (36)	*2 (22)*
MRI	**34 (81)**	**11 (79)**	5 (56)
None/only when indicated	*2 (5)*	*2 (14)*	*2 (22)*

^a^Numbers are given as absolute number (percentage). When the total percentage exceeds 100%, multiple answers could be given to a question

^b^For all items in bold, consensus was reached that these are the most suitable options. For all items in italics, this option was considered to have reached disagreement. Some items exceeding 75% or below 25% were not marked, as the nature of the question does not aim for a consensus to be reached on this topic

^c^For this question, the option ‘Other’ was given. For the elbow, two respondents answered skeletal maturity and whether the physis was closed. The ‘ Other’ option was not chosen for the knee or ankle

^d^For this question, answers exceeding 75% or below 25% were not marked to have reached consensus, as this technique is fairly new, which is probably the reason not many respondents used it yet and would not recommend or disregard it

**Fig. 1:**
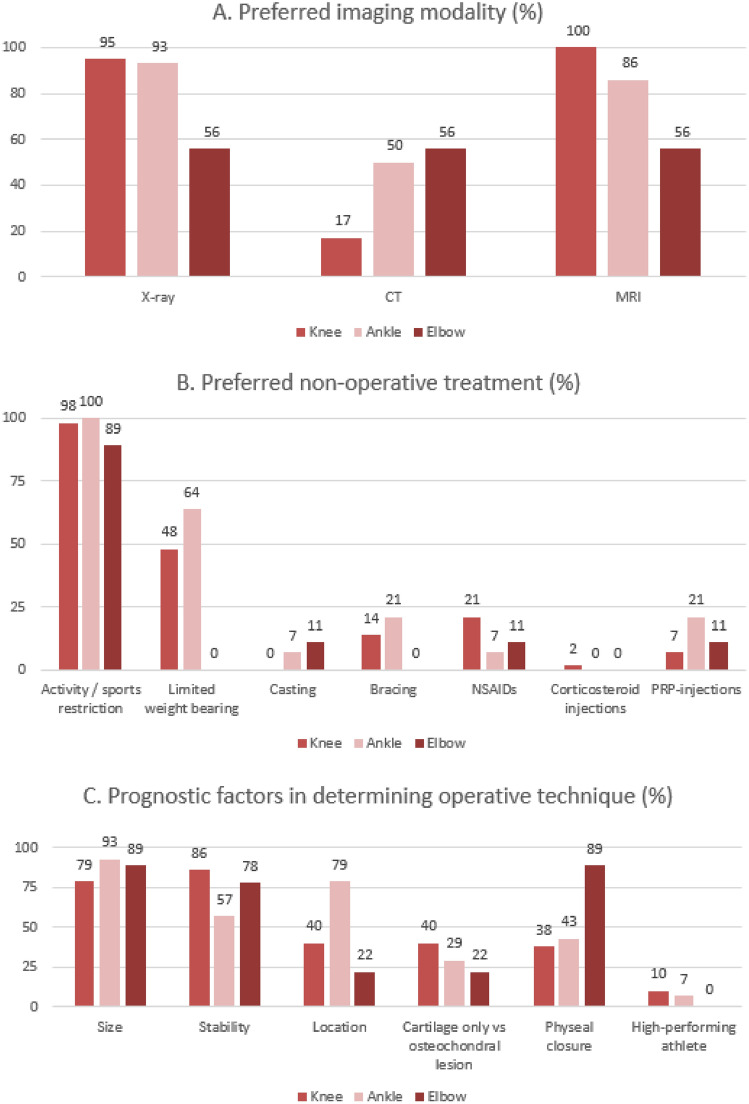
Survey results given in histograms. Numbers given above columns represent percentages of physicians that chose that option

Plain radiography (40 physicians, 95%) and MRI (42 physicians, 100%) are almost always used in the diagnostic work-up of JOCD in the knee (Fig. 1). CT is used by 8 physicians (17%). The physicians who chose to acquire plain radiography, agreed to request an AP and a lateral view. When complaints are unilateral, physicians were divided on whether to request imaging for one knee (22 physicians, 52%) or both knees (20 physicians, 48%). 16 respondents (38%) use a classification system. Examples of classifications that were used included the Clanton and DeLee classification, DiPaola classification, Hefti classification and Nelson classification.

To determine whether a JOCD lesion is stable, respondents almost unanimously use MRI (41 physicians, 98%). Plain radiography (8 physicians, 19%), physical examination (6 physicians, 14%) and CT (3 physicians, 7%) are discouraged for this purpose. Most physicians use imaging of the knee that is already present (27 physicians, 64%) to determine skeletal age, although no method reached consensus.

Activity and/or sports restriction is the non-operative treatment of choice (41 physicians, 98%). The use of non-steroidal anti-inflammatory drugs (NSAIDs), injection therapy through corticosteroids and immobilisation on their own reached consensus not to be used.

Different surgical techniques, including internal fixation with bioabsorbable and non-absorbable devices, antegrade and retrograde drilling, debridement, microfracturing, loose body removal and osteochondral autograft transfer are used. In addition, 8 physicians (20%) mentioned using biologicals, such as platelet-rich plasma (PRP, 6 physicians, 15%) and bone marrow aspirate concentrate (2 physicians, 5%) in the treatment of JOCD.

Consensus was reached that stability (36 physicians, 86%) and size (33 physicians, 79%) of the lesion are the most important prognostic factors in determining the operative technique. Being a high-performing athlete does not influence the operative technique according to respondents. During follow-up after operative treatment, respondents agreed that MRI is the best imaging technique (32 physicians, 76%), although plain radiography was also chosen by 29 physicians (69%). CT was infrequently used (2 physicians, 5%). Most physicians do not record any PROMs. PROMs that were mentioned to be collected were the IKDC score, the Tegner score, the Pedi-IKDC, the KOOS score and the Pedi-FABS score.

### Ankle

Fourteen respondents treated JOCD lesions of the ankle (28%). It was agreed that joint effusion and pain during activities are the most suggestive of OCD in a patient interview. Consensus or disagreement was not reached for the physical examination for any item. Respondents agreed that plain radiography and MRI are the most useful imaging modality. CT was used by 7 respondents (50%).

There was no consensus on whether to perform imaging of one or both ankles when complaints are unilateral. Physicians agreed not to use a classification system for OCD of the ankle. Systems that were mentioned to be used are the Berndt and Harty classification, the Hefti classification and the Ferkel and Sgaglione classification. Consensus was reached that MRI is the way to determine whether a lesion is stable (12 physicians, 86%). Respondents agreed that determining this through plain radiography or physical examination alone is not appropriate. 13 physicians (93%) did not use any genetic testing.

Regarding non-operative treatment for JOCD of the ankle, respondents unanimously use activity and/or sports restriction. Respondents agreed that any form of immobilisation, injection therapy or the use of NSAIDs on their own are not an appropriate treatment. Many different operative techniques are used to treat JOCD of the ankle, of which debridement, microfracturing, drilling and loose body removal were mentioned the most. In addition, 3 physicians (21%) use PRP and 2 (14%) use bone marrow aspirate concentrate in their treatment. It was agreed that the most important prognostic factors in determining the operative technique are size and location of the lesion and that the level of the athlete did not influence the operative technique.

MRI was the preferred imaging modality for follow-up after operative treatment. Not using any imaging as follow-up was discouraged by respondents. No consensus was reached on how long to follow up on a patient after treatment. 10 respondents did not use any PROMs (71%), outcome measures that were recorded were the FAOS, AOFAS and FAAM score.

### Elbow

Out of fifty respondents, nine answered that they treat JOCD lesions of the elbow (18%). Pain on the radial side of the elbow was unanimously the most characteristic finding in the patient interview. Findings that were agreed on not being indicative of JOCD of the elbow are a history of trauma, instability, pain at night, pain on the ulnar side of the elbow and pain only during activities.

No consensus was reached on characteristic findings during physical examination or the use of an imaging modality in the diagnostic process. There was no consensus on whether to perform imaging of one or both elbows when complaints are unilateral. 6 respondents do not use a classification system (67%), the ones that do use the Minami classification. There was no consensus on a method to determine the stability of the lesion, although most (6 physicians, 67%) use MRI. To assess skeletal age, 6 physicians (67%) use the imaging already present, but no consensus was reached. 8 physicians do not use genetic testing (89%), 1 (11%) uses a SMAD3 mutation test in specific cases.

Agreement was reached that activity and/or sports restriction is the preferred non-operative treatment (8 physicians, 89%). Any other method of non-operative treatment is not recommended by the respondents. Operative treatment options that were used the most are debridement, loose body removal and microfracturing. It was agreed that the size of the lesion, aspect of the physes and stability of the lesion are the most important prognostic factors in determining the operative technique. Location of the lesion, whether the lesion is osteochondral or chondral only and being a high-performing athlete are not deemed to be of prognostic value. There was no consensus on the best imaging technique during follow-up. 6 physicians do not use any PROMs (67%), the ones that do use the Oxford Elbow score (3 physicians, 33%) and the Mayo Elbow Performance score (1 physician, 11%).

## Discussion

The most important finding of this study is that physician preferences are diverse across joints. This shows that JOCD has many entities and treatment options. It also shows that there is a lack of consensus between orthopaedic surgeons when it comes to the optimal diagnostic and treatment process. Nevertheless, similarities and agreements can definitely be found. Physicians agreed that the preferred non-operative method for JOCD is activity and/or sports restriction. Furthermore, plain radiography and MRI are the preferred imaging modalities for the knee and ankle, but not the elbow, for which no consensus was reached on any imaging modality. For determining the operative technique, physicians agreed that size of the lesion is an important prognostic factor in all joints.

To our knowledge, a study that explores the diagnosis and treatment preferences for juvenile osteochondritis dissecans for the elbow, knee or ankle has not been performed before. There are multiple guidelines and reviews on the optimal treatment of osteochondritis dissecans, but how these are implemented in everyday practice has not been investigated before. Guidelines on treating juvenile osteochondritis dissecans are scarcer than those of adult OCD, so gathering expert opinion on this form of OCD is even more valuable.

Forty-two out of fifty respondents treated JOCD of the knee, which was the part of the survey that was filled out most often. This supports the literature that the knee joint is most affected [5, 21].

Joint effusion and pain during activities were considered to be the most characteristic findings during the patient interview for the knee and ankle. Interestingly, this was not the case for the elbow. Only pain on the radial side of the elbow was considered to be a characteristic finding for this joint, which is also the only suggestive finding for JOCD of the elbow in the beginning stages in the literature [9, 23]. It is interesting to see that these symptoms are all non-specific. It was agreed that a history of trauma, pain at night and instability complaints were not typical for JOCD in any joint.

There were also some interesting findings concerning physical examination. For the knee, the Wilson’s test is a test that if positive, might indicate the presence of OCD [31]. However, only 14% of physicians thought this test to be characteristic of OCD, supporting previous research that the Wilson’s test, although specifically designed for OCD, is of little diagnostic value [7]. Therefore, we recommend not to perform this test. A test that might indicate the presence of OCD in the elbow is the radiocapitellar test. However, its sensitivity and specificity are not well known [10]. Only 33% of physicians in this survey thought this test to be indicative of OCD, which suggests that the clinical value of this test is also low. We also recommend not to perform this test.

Plain radiography and MRI were considered the preferred imaging modalities for the knee and ankle. Consensus was reached that CT is not the best imaging technique for diagnosing JOCD of the knee.

For the elbow, however, no consensus was reached for any imaging modality. Half or more of the physicians in the elbow and ankle group also routinely use CT. Previous research has shown that MRI is the imaging modality of choice over CT for OCD of the knee [15, 17, 20]. For the elbow, however, there is little research to prefer one over the other, although multiple studies have suggested that CT is more sensitive in detecting OCDs of the elbow [26]. For the ankle, despite there being a preference for MRI over CT in this survey, there is no convincing evidence in literature that prefers MRI over CT or vice versa, as both techniques have advantages and disadvantages and have a similar sensitivity and specificity in diagnosing JOCD [25, 27, 29].

Plain radiography is recommended as a screening instrument for JOCD of the knee, ankle and elbow. For the knee, if JOCD is still suspected after a normal radiograph, we recommend performing an MRI scan. For the elbow and ankle, evidence for CT or MRI is not as conclusive, as they both have advantages and disadvantages. We also recommend using MRI for pre-operative planning and post-operative follow-up for the knee. Again, evidence for the elbow and ankle is not as conclusive for pre-operative planning and post-operative follow-up.

Activity and/or sports restriction was the preferred non-operative treatment in all joints. These measures on their own tend to be a relatively successful treatment for JOCD in the beginning stages [13, 22]. All other treatment options, except for limited weight bearing, reached disagreement, meaning that on their own, these treatments are discouraged. This concurs with the literature, which shows that non-operative measures other than activity and/or sports restriction, such as immobilisation and injection therapy, are of little therapeutic value [1, 19]. Therefore, we recommend that activity and/or sports restriction should be the only non-operative treatment for JOCD, unless additional factors indicate different treatment.

Across the different joints, agreement was reached that size of the lesion is an important prognostic factor in determining the operative technique. Additionally, stability was deemed an important prognostic factor for the elbow and knee. Size, stability and location of the lesion have proved to be important prognostic factors for treatment of OCD of the knee in previous research [2, 16]. Another important prognostic factor for the elbow was physeal closure, which was not the case for the knee or ankle. Finally, it was agreed that being a high-performing athlete does not influence the operative technique for any joint.

Studies have shown that a capitellar OCD in a patient with closed physes has worse outcomes than those with open physes. This trend has also been described for OCDs of the knee and ankle, but did not come forward as much in this survey [5, 8, 13]. Location of the lesion was deemed to be an important prognostic factor in the ankle, although its prognostic value remains unclear in literature [6, 12, 28].

Despite spreading this survey through numerous media, this study was limited by its sample size of 50 physicians. With more respondents, a more representative view of orthopaedic surgeons’ preferences could have been given and hard conclusions could have been drawn. However, it still provides valuable insight into the preferences of orthopaedic surgeons when it comes to treating this relatively rare condition. Another limitation is that because of the many different treatment options that are used and the many different severities JOCD can present itself in, it is hard to distinguish which method is best in which situation. We tried to tackle this problem using cases, but answers were still very diverse and challenging to compare between joints (see supplementary material).

In conclusion, this survey was conducted to assess physicians’ current diagnosis and treatment preferences in different countries for juvenile osteochondritis dissecans of the knee, ankle and elbow. Results were diverse across joints, but generally aligned with current knowledge. These findings help us to understand how research on juvenile osteochondritis dissecans is applied in current practice. The information that was gained through this survey can be used towards future studies on developing a diagnostic and treatment algorithm for JOCD of the elbow, knee and ankle.

## Conclusion

Activity and/or sports restriction was the non-operative treatment of choice. Furthermore, plain radiography and MRI were the preferred imaging modalities for the knee and ankle, but not for the elbow. For determining the operative technique, physicians agreed that the size of the lesion is an important prognostic factor in all joints. These findings help us understand how juvenile osteochondritis dissecans is treated in current practice and may provide opportunities for improvement.

### Supplementary Information

Below is the link to the electronic supplementary material.

Supplementary file1 (PDF 554 KB)

Supplementary file2 (XLSX 42 KB)

## Data Availability

Data was supplied in the supplementary material.

## Abbreviations

ESSKA: European Society of Sports Traumatology, Knee Surgery and Arthroscopy
JOCD: Juvenile osteochondritis dissecans
NSAID: Non-steroidal anti-inflammatory drug
OCD: Osteochondritis dissecans
PROM: Patient-reported outcome measure
PRP: Platelet-rich plasma

## Declarations

### Social media handles

LinkedIN: Laura Kemmeren, Christiaan van Bergen, Max Reijman, Tom Piscaer.
