# Supplementary material for: Physician preferences in diagnostics and treatment of juvenile osteochondritis dissecans are diverse across the knee, ankle and elbow: an ESSKA survey
Source: Knee Surg Sports Traumatol Arthrosc. 2023 Oct 3;31(11):5228–37. doi: 10.1007/s00167-023-07563-0 (PMC10598091; doi:10.1007/s00167-023-07563-0)

Appendix 1: survey

Diagnosis and treatment of juvenile osteochondritis dissecans (JOCD)

Section 1 - Demographics

1.What is your current profession?

- Orthopaedic surgeon
- Orthopaedic surgeon in training
- Trauma surgeon
- Trauma surgeon in training
- Sports physician
- Sports physician in training
- Other

2.How many years of experience do you have within your current profession?

- I am currently in residency
- 0-5 years since residency
- 5-10 years since residency
- 10-15 years since residency
- 15-20 years since residency
- 20+ years since residency

3.Approximately how many JOCD lesions do you treat annually, both operatively and non-operatively?

- 1-10
- 10-20
- 20-50
- 50+

4.Which factor is key to you in classifying osteochondritis dissecans as juvenile?

- Age
- Skeletal maturity
- Both

Section 2 - Elbow

5.Do you treat JOCD lesions of the elbow?

- Yes → question 6
- No → question 26

6.Approximately how many JOCD lesions of the elbow do you treat annually, both operatively and non-operatively?

- 1-5
- 5-10
- 10-15
- 20+

7.Which of the following aspects during the patient interview makes you suspect a JOCD of the elbow the most? (Choose a maximum of 3 aspects)

- ☐ History of trauma

- ☐ Locking complaint
- ☐ Instability complaints
- ☐ Pain at night
- ☐ Pain on radial side of elbow
- ☐ Pain on ulnar side of elbow
- ☐ Pain only during activities
- ☐ Participation in a throwing sport
- ☐ Other

8.Which of the following aspects during the physical examination are most important to you, if you suspect a JOCD of the elbow? (Choose a maximum of 3 aspects)

- ☐ Presence of joint effusion
- ☐ Point tenderness on palpation
- ☐ Presence of crepitus
- ☐ Limited range of motion
- ☐ Positive radiocapitellar test
- ☐ Other

9.If you suspect a patient of having JOCD of the elbow, which imaging modality or modalities do you usually acquire? (multiple answers may be chosen)

- ☐ X-ray
- ☐ Ultrasound
- ☐ CT
- ☐ CT-arthrography
- ☐ SPECT
- ☐ MRI
- ☐ MR-arthrography
- ☐ Bone scintigraphy
- ☐ None
- ☐ Other

10.If you selected "X-ray" in the previous question, which of the following views do you usually request?

- ☐ AP view with elbow in extension
- ☐ AP view with elbow in 45˚ flexion
- ☐ Lateral view
- ☐ External oblique view
- ☐ Internal oblique view
- ☐ Radial head view
- ☐ Other

11.When you suspect a patient of having JOCD of their elbow do you usually perform imaging of one or both elbows?

- o One elbow
- o Both elbows

12.Which classification system do you usually use to grade a JOCD of the elbow?

- o Minami classification
- o Itsubo classification
- o I do not use any classification system

- Other

13.How do you determine whether a JOCD lesion is stable or unstable? (multiple answers may be chosen)

- ☐ History of patient
- ☐ Physical examination
- ☐ Range of motion of the elbow
- ☐ X-ray
- ☐ CT
- ☐ MRI
- ☐ Arthroscopically
- ☐ Other

14.Do you assess skeletal age during the diagnostic process of a JOCD of the elbow and if so, how do you do this? (multiple answers may be chosen)

- ☐ No, I do not
- ☐ Yes, through the Tanner stage
- ☐ Yes, through the imaging of the elbow that is already present
- ☐ Yes, through an additional X-ray of the hand

15.Do you perform any genetic tests after diagnosing a JOCD of the elbow?

- No, I do not
- Yes, I perform a SMAD3 mutation test in specific cases
- Yes, I always perfrom a SMAD3 mutation test
- Yes, I refer patients to a clinical geneticist in specific cases
- Yes, I always refer patients to a clinical geneticist
- Other

16.What is your preferred non-operative treatment plan for JOCD lesions of the elbow? (choose a maximum of 3 options)

- ☐ Activity restriction/sports restriction
- ☐ Immoblization through bracing
- ☐ Immobilization through casting
- ☐ Injection therapy through corticosteroids
- ☐ Injection therapy through platelet-rich plasma
- ☐ Non-steroidal anti-inflammatory drugs
- ☐ Other

17.After what time period of conservative treatment do you usually resort to operative treatment in a radiologically stable lesion in a boy with a skeletal age of 12 with persistent pain and no changes on radiography?

- 0 months
- 3 months
- 6 months
- 9 months
- 12 months
- 18 months
- 24 months
- I usually do not treat a radiologically stable lesion operatively
- Other

18.Which type(s) of operative treatment do you use for JOCD lesions of the elbow? (multiple answers may be chosen)

- ☐ Internal fixation with non-absorbable devices
- ☐ Internal fixation with bioabsorbable devices
- ☐ Bone grafting
- ☐ Drilling with a Kirschner wire
- ☐ Retrograde drilling
- ☐ Debridement
- ☐ Microfracturing
- ☐ Autologous chondrocyte implantation
- ☐ Osteochondral autograft plugs
- ☐ Osteochondral allograft
- ☐ Loose body removal
- ☐ Other

19.Do you use biologicals in the treatment of JOCD of the elbow and if so, which?

- o No
- o Yes, platelet rich plasma (PRP)
- o Yes, bone marrow aspirate concentrate (BMAC)
- o Other

20.What are the most important prognostic factors to you in determining the operative technique for a JOCD of the elbow? (choose a maximum of 3 options)

- ☐ Size of the lesion
- ☐ Stability of the lesion
- ☐ Location of the lesion
- ☐ Lesion of cartilage only vs osteochondral lesion
- ☐ Whether the physis has closed
- ☐ High performing athlete

21.What is your preferred treatment for the following patient? (a combination of different techniques may be chosen that you would apply together)

A boy, skeletal age 13, plays baseball two times a week and has pain in his right elbow (dominant side). Newly diagnosed with a primary OCD of 12mm of his capitellum. The lesion is radiologically unstable (articular cartilage is breached and there is fluid behind the subchondral bone on MRI) but not displaced.

- ☐ Immobilization through bracing
- ☐ Immobilization through casting
- ☐ Non-steroidal anti-inflammatory drugs
- ☐ Injection therapy through corticosteroids
- ☐ Injection therapy through platelet-rich plasma
- ☐ Activity restriction/sports restriction
- ☐ Internal fixation with non-absorbable devices
- ☐ Internal fixation with bioabsorbable devices
- ☐ Bone grafting
- ☐ Antegrade drilling
- ☐ Retrograde drilling
- ☐ Debridement
- ☐ Microfracturing
- ☐ Autologous chondrocyte implantation

- ☐ Osteochondral autograft plugs
- ☐ Osteochondral allograft
- ☐ Loose body removal
- ☐ Other

22.What is your preferred treatment for the following patient? (a combination of different techniques may be chosen that you would apply together)

A girl, skeletal age 11, does gymnastics three times a week and has pain in her right elbow (dominant side). Diagnosed with a primary OCD of 12mm of her capitellum six months ago. Sports restriction did not help. The lesion is radiologically stable (articular cartilage is breached and there is a low signal rim behind the fragment on MRI). There are no relevant changes on radiography, when comparing with the first images of six months ago.

- ☐ Activity restriction/sports restriction
- ☐ Immobilization through bracing
- ☐ Immobilization through casting
- ☐ Injection therapy through corticosteroids
- ☐ Injection therapy through platelet-rich plasma
- ☐ Non-steroidal anti-inflammatory drugs
- ☐ Internal fixation with non-absorbable devices
- ☐ Internal fixation with bioabsorbable devices
- ☐ Bone grafting
- ☐ Antegrade drilling
- ☐ Retrograde drilling
- ☐ Debridement
- ☐ Microfracturing
- ☐ Autologous chondrocyte implantation
- ☐ Osteochondral autograft plugs
- ☐ Osteochondral allograft
- ☐ Loose body removal
- ☐ Other

23.Which imaging modalities do you usually perform during follow-up after operative treatment for JOCD of the elbow? (multiple answers may be chosen)

- ☐ X-ray
- ☐ Ultrasound
- ☐ CT
- ☐ CT-arthrography
- ☐ SPECT
- ☐ MRI
- ☐ MR-arthrography
- ☐ None/only when indicated
- ☐ Other

24.Over what time period do you schedule follow-up visits after operative treatment of JOCD of the elbow?

- ○ 1 year
- ○ 2 years
- ○ 3 years
- ○ Until the end of growth
- ○ Until end of symptoms

- Other

25.Do you record any patient reported outcome measures (PROMs) after treatment for JOCD of the elbow? (multiple answers may be chosen)

- ☐ Yes, the Mayo Elbow Performance score
- ☐ Yes, the Oxford Elbow score
- ☐ No
- ☐ Other

Section 3 - Hip

26.Do you treat JOCD lesions of the hip?

- Yes → question 27
- No → question 47

27.Approximately how many JOCD lesions of the hip do you treat annually, both operatively and non-operatively?

- 1-5
- 5-10
- 10-15
- 20+

28.Which of the following aspects during the patient interview makes you suspect a JOCD of the hip the most? (Choose a maximum of 3 aspects)

- ☐ Clicking of the hip
- ☐ History of trauma
- ☐ Instability complaints
- ☐ Joint effusion
- ☐ Limited range of motion
- ☐ Locking of the hip
- ☐ Pain at night
- ☐ Pain during activities
- ☐ Other

29.Which of the following aspects during the physical examination are most important to you, if you suspect a JOCD of the hip? (Choose a maximum of 3 aspects)

- ☐ Abnormal gait
- ☐ Limited range of motion
- ☐ Pain on passive mobilisation
- ☐ Positive FADIR test (flexion, adduction, internal rotation)
- ☐ Positive FABER test (flexion, abduction, external rotation)
- ☐ Other

30.If you suspect a patient of having JOCD of the hip, which imaging modality or modalities do you usually acquire? (multiple answers may be chosen)

- ☐ X-ray
- ☐ Ultrasound
- ☐ CT
- ☐ CT-arthrography
- ☐ SPECT

- ☐ MRI
- ☐ MR-arthrography
- ☐ Bone scintigraphy
- ☐ None
- ☐ Other

31.If you selected "X-ray" in the previous question, which of the following views do you usually request? (multiple answers may be chosen)

- ☐ AP view
- ☐ Axial view
- ☐ Lateral view
- ☐ Horizontal beam lateral view
- ☐ Dunn view
- ☐ Lauenstein view
- ☐ Other

32.When you suspect a patient of having JOCD of their hip do you usually perform imaging of one or both hips?

- o One hip
- o Both hips

33.Which classification system do you usally use to grade a JOCD of the hip?

- o Anderson classification
- o Clanton and DeLee classification
- o DiPaola classification
- o Hefti classification
- o Nelson classification
- o I do not use any classification system
- o Other

34.How do you determine whether a JOCD lesion is stable or unstable? (multiple answers may be chosen)

- ☐ History of patient
- ☐ Physical examination
- ☐ Range of motion of the hip
- ☐ X-ray
- ☐ CT
- ☐ MRI
- ☐ Arthroscopically
- ☐ Other

35.Do you assess skeletal age during the diagnostic process of a JOCD of the hip and if so, how do you do this? (multiple answers may be chosen)

- ☐ No, I do not
- ☐ Yes, through the Tanner stage
- ☐ Yes, through the imaging of the hip that is already present
- ☐ Yes, through an additional X-ray of the hand

36.Do you perform any genetic tests after diagnosing a JOCD of the hip?

- o No, I do not

- Yes, I perform a SMAD3 mutation test in specific cases
- Yes, I always perform a SMAD3 mutation test
- Yes, I refer patients to a clinical geneticist in specific cases
- Yes, I always refer patients to a clinical geneticist
- Other

37.What is your preferred non-operative treatment plan for JOCD lesions of the hip? (choose a maximum of 3 options)

- ☐ Activity restriction/sports restriction
- ☐ Immoblization through bracing
- ☐ Immobilization through casting
- ☐ Injection therapy through corticosteroids
- ☐ Injection therapy through platelet-rich plasma
- ☐ Limited weight bearing
- ☐ Non-steroidal anti-inflammatory drugs
- ☐ Other

38.After what time period of conservative treatment do you usually resort to operative treatment in a radiologically stable lesion in a boy with a skeletal age of 12 with persistent pain and no changes on radiography?

- 0 months
- 3 months
- 6 months
- 9 months
- 12 months
- 18 months
- 24 months
- I usually do not treat a radiologically stable lesion operatively
- Other

39.Which type(s) of operative treatment do you use for JOCD lesions of the hip? (multiple answers may be chosen)

- ☐ Internal fixation with non-absorbable devices
- ☐ Internal fixation with bioabsorbable devices
- ☐ Bone grafting
- ☐ Drilling
- ☐ Retrograde drilling
- ☐ Debridement
- ☐ Microfracturing
- ☐ Autologous chondrocyte implantation
- ☐ Osteochondral autograft plugs
- ☐ Osteochondral allograft
- ☐ Loose body removal
- ☐ Intertrochanteric osteotomy
- ☐ Other

40.Do you use biologicals in the treatment of JOCD of the hip and if so, which?

- No
- Yes, platelet rich plasma (PRP)
- Yes, bone marrow aspirate concentrate (BMAC)
- Other

41.What are the most important prognostic factors to you in determining the operative technique for a JOCD of the hip? (choose a maximum of 3 options)

- ☐ Size of the lesion
- ☐ Stability of the lesion
- ☐ Location of the lesion
- ☐ Lesion of cartilage only vs osteochondral lesion
- ☐ Whether the physis has closed
- ☐ High performing athlete

42.What is your preferred treatment for the following patient? (a combination of different techniques may be chosen that you would apply together)

A boy, skeletal age 13, plays football two times a week and has pain in his right groin. Newly diagnosed with a primary OCD lesion of 12mm near the fovea of the right hip. The lesion is radiologically unstable (articular cartilage is breached and there is fluid behind the subchondral bone on MRI) but not displaced.

- ☐ Activity restriction
- ☐ Injection therapy through corticosteroids
- ☐ Injection therapy through platelet-rich plasma
- ☐ Limited weight bearing
- ☐ Internal fixation with non-absorbable devices
- ☐ Internal fixation with bioabsorbable devices
- ☐ Bone grafting
- ☐ Drilling
- ☐ Retrograde drilling
- ☐ Debridement
- ☐ Microfracturing
- ☐ Autologous chondrocyte implantation
- ☐ Osteochondral autograft plugs
- ☐ Osteochondral allograft
- ☐ Loose body removal
- ☐ Intertrochanteric osteotomy
- ☐ Other

43.What is your preferred treatment for the following patient? (a combination of different techniques may be chosen that you would apply together)

A girl, skeletal age 11, runs three times a week and has pain in her right groin. Diagnosed with a primary OCD lesion of 12mm near the fovea of the right hip six months ago. Sports restriction did not help. The lesion is radiologically stable (articular cartilage is breached and there is a low signal rim behind the fragment on MRI). There are no relevant changes on radiography, when comparing with the first images of six months ago.

- ☐ Activity restriction
- ☐ Injection therapy through corticosteroids
- ☐ Injection therapy through platelet-rich plasma
- ☐ Limited weight bearing
- ☐ Internal fixation with non-absorbable devices
- ☐ Internal fixation with bioabsorbable devices
- ☐ Bone grafting
- ☐ Antegrade drilling
- ☐ Retrograde drilling

- ☐ Debridement
- ☐ Microfracturing
- ☐ Autologous chondrocyte implantation
- ☐ Osteochondral autograft plugs
- ☐ Osteochondral allograft
- ☐ Loose body removal
- ☐ Intertrochanteric osteotomy
- ☐ Other

44.Which imaging modalities do you usually perform during follow-up after operative treatment for JOCD of the hip? (multiple answers may be chosen)

- ☐ X-ray
- ☐ Ultrasound
- ☐ CT
- ☐ CT-arthrography
- ☐ SPECT
- ☐ MRI
- ☐ MR-arthrography
- ☐ None/only when indicated
- ☐ Other

45.Over what time period do you schedule follow-up visits after operative treatment of JOCD of the hip?

- ☐ 1 year
- ☐ 2 years
- ☐ 3 years
- ☐ Until the end of growth
- ☐ Until free of symptoms
- ☐ Other

46.Do you record any patient reported outcome measures (PROMs) after treatment for JOCD of the hip? (multiple answers may be chosen)

- ☐ Yes, the HAGOS score
- ☐ Yes, the Harris hip score
- ☐ Yes, the HOOS score
- ☐ Yes, the IHOT33
- ☐ Yes, the Oxford hip score
- ☐ No
- ☐ Other

Section 4 - Knee

47.Do you treat JOCD lesions of the knee?

- o Yes → question 48
- o No → question 69

48.Approximately how many JOCD lesions of the knee do you treat annually, both operatively and non-operatively?

- o 1-5
- o 5-10

- 10-15
- 20+

49.Which of the following aspects during the patient interview makes you suspect a JOCD of the knee the most? (Choose a maximum of 3 aspects)

- ☐ Clicking of the knee
- ☐ History of trauma
- ☐ Instability complaints
- ☐ Joint effusion
- ☐ Limited range of motion
- ☐ Locking of the knee
- ☐ Pain at night
- ☐ Pain during activities
- ☐ Other

50.Which of the following aspects during the physical examination are most important to you, if you suspect a JOCD of the knee? (Choose a maximum of 3 aspects)

- ☐ Abnormal gait pattern
- ☐ Limited range of motion
- ☐ Limited stability
- ☐ Presence of joint effusion
- ☐ Point tenderness on palpation
- ☐ Positive Wilson's test
- ☐ Presence of crepitus
- ☐ Other

51.If you suspect a patient of having JOCD of the knee, which imaging modality or modalities do you usually acquire? (multiple answers may be chosen)

- ☐ X-ray
- ☐ Ultrasound
- ☐ CT
- ☐ CT-arthrography
- ☐ SPECT
- ☐ MRI
- ☐ MR-arthrography
- ☐ Bone scintigraphy
- ☐ None
- ☐ Other

52.If you selected "X-ray" in the previous question, which of the following views do you usually request? (multiple answers may be chosen)

- ☐ AP view
- ☐ Lateral view
- ☐ Rosenberg view
- ☐ Sunrise view
- ☐ Tunnel view
- ☐ Whole leg X-ray
- ☐ Other

53.When you suspect a patient of having JOCD, do you usually perform imaging of one or both knees?

- One knee
- Both knees

54.Which classification system do you usually use to grade a JOCD of the knee?

- Clanton and DeLee classification
- DiPaola classification
- Hefti classification
- Nelson classification
- I do not use any classification system
- Other

55.How do you determine whether a JOCD lesion is stable or unstable? (multiple answers may be chosen)

- ☐ History of patient
- ☐ Physical examination
- ☐ Range of motion of the knee
- ☐ X-ray
- ☐ CT
- ☐ MRI
- ☐ Arthroscopically
- ☐ Other

56.Do you assess skeletal age during the diagnostic process of a JOCD of the knee and if so, how do you do this? (multiple answers may be chosen)

- ☐ No, I do not
- ☐ Yes, through the Tanner stage
- ☐ Yes, through the imaging of the knee that is already present
- ☐ Yes, through an additional X-ray of the hand

57.Do you perform any genetic tests after diagnosing a JOCD of the knee?

- No, I do not
- Yes, I perform a SMAD3 mutation test in specific cases
- Yes, I always perform a SMAD3 mutation test
- Yes, I refer patients to a clinical geneticist in specific cases
- Yes, I always refer patients to a clinical geneticist
- Other

58.What is your preferred non-operative treatment plan for JOCD lesions of the knee? (choose a maximum of 3 options)

- ☐ Activity restriction/sports restriction
- ☐ Immobilization through bracing
- ☐ Immobilization through casting
- ☐ Immobilization through splinting
- ☐ Injection therapy through corticosteroids
- ☐ Injection therapy through platelet-rich plasma
- ☐ Limited weight bearing
- ☐ Non-steroidal anti-inflammatory drugs
- ☐ Other

59.After what time period of conservative treatment do you usually resort to operative treatment in a radiologically stable lesion in a boy with a skeletal age of 12 with persistent pain and no changes on radiography?

- 0 months
- 3 months
- 6 months
- 9 months
- 12 months
- 18 months
- 24 months
- I usually do not treat a radiologically stable lesion operatively
- Other

60.Which type(s) of operative treatment do you use for JOCD lesions of the knee? (multiple answers may be chosen)

- ☐ Antegrade drilling
- ☐ Retrograde drilling
- ☐ Notch drilling
- ☐ Transarticular drilling
- ☐ Internal fixation with non-absorbable devices
- ☐ Internal fixation with bioabsorbable devices
- ☐ Bone grafting
- ☐ Debridement
- ☐ Microfracturing
- ☐ Autologous chondrocyte implantation
- ☐ Osteochondral autograft plugs
- ☐ Osteochondral allograft
- ☐ Loose body removal
- ☐ Other

61.Do you use biologicals in the treatment of JOCD of the knee and if so, which?

- No
- Yes, platelet rich plasma (PRP)
- Yes, bone marrow aspirate concentrate (BMAC)
- Other

62.What are the most important prognostic factors to you in determining the operative technique for a JOCD of the knee? (choose a maximum of 3 options)

- ☐ Size of the lesion
- ☐ Stability of the lesion
- ☐ Location of the lesion
- ☐ Lesion of cartilage only vs osteochondral lesion
- ☐ Whether the physis has closed
- ☐ High performing athlete

63.What is your preferred treatment for the following patient? (a combination of different techniques may be chosen that you would apply together)

A boy, skeletal age 13, plays football two times a week and has pain in his right knee. Newly diagnosed with a primary OCD lesion of 12mm of his medial femoral condyle on the right side. The

lesion is radiologically unstable (articular cartilage is breached and there is fluid behind the subchondral bone on MRI) but not displaced.

- ☐ Immobilization through casting
- ☐ Immobilization through bracing
- ☐ Immobilization through splinting
- ☐ Limited weight bearing
- ☐ Activity restriction
- ☐ Injection therapy through corticosteroids
- ☐ Injection therapy through platelet-rich plasma
- ☐ Antegrade drilling
- ☐ Transarticular drilling
- ☐ Retroarticular drilling
- ☐ Notch drilling
- ☐ Internal fixation with non-absorbable devices
- ☐ Internal fixation with bioabsorbable devices
- ☐ Bone grafting
- ☐ Debridement
- ☐ Microfracturing
- ☐ Autologous chondrocyte implantation
- ☐ Osteochondral autograft plugs
- ☐ Osteochondral allograft
- ☐ Loose body removal
- ☐ Other

64.What is your preferred treatment for the following patient? (a combination of different techniques may be chosen that you would apply together)

A girl, skeletal age 11, plays hockey three times a week and has pain in her right knee. Diagnosed with a primary OCD lesion of 12mm of her right medial femoral condyle six months ago. Sports restriction did not help. The lesion is radiologically stable (articular cartilage is breached and there is a low signal rim behind the fragment on MRI). There are no relevant changes on radiography, when comparing with the first images of six months ago.

- ☐ Immobilization through casting
- ☐ Immobilization through bracing
- ☐ Immobilization through splinting
- ☐ Limited weight bearing
- ☐ Activity restriction
- ☐ Injection therapy through corticosteroids
- ☐ Injection therapy through platelet-rich plasma
- ☐ Antegrade drilling
- ☐ Transarticular drilling
- ☐ Retroarticular drilling
- ☐ Notch drilling
- ☐ Internal fixation with non-absorbable devices
- ☐ Internal fixation with bioabsorbable devices
- ☐ Bone grafting
- ☐ Debridement
- ☐ Microfracturing
- ☐ Autologous chondrocyte implantation
- ☐ Osteochondral autograft plugs
- ☐ Osteochondral allograft
- ☐ Loose body removal

☐ Other

65.Which imaging modalities do you usually perform during follow-up after operative treatment for JOCD of the knee? (multiple answers may be chosen)

☐ X-ray
☐ Ultrasound
☐ CT
☐ CT-arthrography
☐ SPECT
☐ MRI
☐ MR-arthrography
☐ None/only when indicated
☐ Other

66.Over what time period do you schedule follow-up visits after operative treatment of JOCD of the knee?

- 1 year
- 2 years
- 3 years
- Until the end of growth
- Other

67.Do you record any patient reported outcome measures (PROMs) after treatment for JOCD of the knee? (multiple answers may be chosen)

☐ Yes, the IKDC score
☐ Yes, the IKDC KIDS
☐ Yes, the KOOS score
☐ Yes, the PEDIFABS
☐ Yes, the Tegner score
☐ No
☐ Other

Section 5 - Ankle

68.Do you treat JOCD lesions of the ankle?

- Yes → question 69
- No → end

69.Approximately how many JOCD lesions of the ankle do you treat annually, both operatively and non-operatively?

- 1-5
- 5-10
- 10-15
- 20+

70.Which of the following aspects during the patient interview makes you suspect a JOCD of the ankle the most? (Choose a maximum of 3 aspects)

☐ Clicking of the ankle
☐ History of trauma
☐ Instability complaints

- ☐ Joint effusion
- ☐ Limited range of motion
- ☐ Locking of the ankle
- ☐ Pain during activity
- ☐ Pain at night
- ☐ Other

71.Which of the following aspects during the physical examination are most important to you, if you suspect a JOCD of the ankle? (Choose a maximum of 3 aspects)

- ☐ Ligamentous laxity
- ☐ Limited range of motion
- ☐ Presence of crepitus
- ☐ Presence of joint effusion
- ☐ Point tenderness on palpation
- ☐ Other

72.If you suspect a patient of having JOCD of the ankle, which imaging modality or modalities do you usually acquire? (multiple answers may be chosen)

- ☐ X-ray
- ☐ Ultrasound
- ☐ CT
- ☐ CT-arthrography
- ☐ SPECT
- ☐ MRI
- ☐ MR-arthrography
- ☐ Bone scintigraphy
- ☐ None
- ☐ Other

73.If you selected "X-ray" in the previous question, which of the following views do you usually request? (multiple answers may be chosen)

- ☐ AP view
- ☐ Lateral view
- ☐ Mortise view
- ☐ Heel rise view
- ☐ Horizontal beam lateral view
- ☐ Other

74.When you suspect a patient of having JOCD, do you usually perform imaging of one or both ankles?

- o One ankle
- o Both ankles

75.Which classification system do you usually use to grade a JOCD of the ankle?

- o Berndt and Harty classification
- o DiPaola classification
- o Hefti classification
- o Hepple classification
- o Ferkel and Sgaglione classification
- o I do not use any classification system

- Other

76.How do you determine whether a JOCD lesion is stable or unstable? (multiple answers may be chosen)

- ☐ History of patient
- ☐ Physical examination
- ☐ Range of motion of the ankle
- ☐ X-ray
- ☐ CT
- ☐ MRI
- ☐ Arthroscopically
- ☐ Other

77.Do you perform any genetic tests after diagnosing a JOCD of the ankle?

- No, I do not
- Yes, I perform a SMAD3 mutation test in specific cases
- Yes, I always perform a SMAD3 mutation test
- Yes, I refer patients to a clinical geneticist in specific cases
- Yes, I always refer patients to a clinical geneticist
- Other

78.What is your preferred non-operative treatment plan for JOCD lesions of the ankle? (choose a maximum of 3 options)

- ☐ Activity restriction/sports restriction
- ☐ Immobilization through bracing
- ☐ Immobilization through casting
- ☐ Immobilization through splinting
- ☐ Injection therapy through corticosteroids
- ☐ Injection therapy through platelet-rich plasma
- ☐ Limited weight bearing
- ☐ Non-steroidal anti-inflammatory drugs
- ☐ Other

79.After what time period of conservative treatment do you usually resort to operative treatment in a radiologically stable lesion in a boy with a skeletal age of 12 with persistent pain and no changes on radiography?

- 0 months
- 3 months
- 6 months
- 9 months
- 12 months
- 18 months
- 24 months
- I usually do not treat a radiologically stable lesion operatively
- Other

80.Which type(s) of operative treatment do you use for JOCD lesions of the ankle? (multiple answers may be chosen)

- ☐ Drilling
- ☐ Internal fixation with non-absorbable devices

- ☐ Internal fixation with bioabsorbable devices
- ☐ Bone grafting
- ☐ Debridement
- ☐ Microfracturing
- ☐ Autologous chondrocyte implantation
- ☐ Osteochondral autograft plugs
- ☐ Osteochondral allograft
- ☐ Loose body removal
- ☐ Other

81.Do you use biologicals in the treatment of JOCD of the ankle and if so, which?

- o No
- o Yes, platelet rich plasma (PRP)
- o Yes, bone marrow aspirate concentrate (BMAC)
- o Other

82.What are the most important prognostic factors to you in determining the operative technique for a JOCD of the ankle? (choose a maximum of 3 options)

- ☐ Size of the lesion
- ☐ Stability of the lesion
- ☐ Location of the lesion
- ☐ Lesion of cartilage only vs osteochondral lesion
- ☐ Whether the physis has closed
- ☐ High performing athlete

83.What is your preferred treatment for the following patient? (a combination of different techniques may be chosen that you would apply together)

A boy, skeletal age 13, plays football two times a week and has pain in his right ankle. Newly diagnosed with a primary OCD lesion of 12mm of the medial talar dome on the right side. The lesion is radiologically unstable (articular cartilage is breached and there is fluid behind the subchondral bone on MRI) but not displaced.

- ☐ Immobilization through casting
- ☐ Immobilization through bracing
- ☐ Immobilization through splinting
- ☐ Limited weight bearing
- ☐ Activity restriction
- ☐ Injection therapy through corticosteroids
- ☐ Injection therapy through platelet-rich plasma
- ☐ Internal fixation with non-absorbable devices
- ☐ Internal fixation with bioabsorbable devices
- ☐ Drilling
- ☐ Bone grafting
- ☐ Debridement
- ☐ Microfracturing
- ☐ Autologous chondrocyte implantation
- ☐ Osteochondral autograft plugs
- ☐ Osteochondral allograft
- ☐ Loose body removal
- ☐ Other

84.What is your preferred treatment for the following patient? (a combination of different techniques may be chosen that you would apply together)

A girl, skeletal age 11, plays hockey three times a week and has pain in her right ankle. Diagnosed with a primary OCD lesion of 12mm of the medial talar dome six months ago. Sports restriction did not help. The lesion is radiologically stable (articular cartilage is breached and there is a low signal rim behind the fragment on MRI). There are no relevant changes on radiography, when comparing with the first images of six months ago.

- ☐ Immobilization through casting
- ☐ Immobilization through bracing
- ☐ Immobilization through splinting
- ☐ Limited weight bearing
- ☐ Activity restriction
- ☐ Injection therapy through corticosteroids
- ☐ Injection therapy through platelet-rich plasma
- ☐ Internal fixation with non-absorbable devices
- ☐ Internal fixation with bioabsorbable devices
- ☐ Drilling
- ☐ Bone grafting
- ☐ Debridement
- ☐ Microfracturing
- ☐ Autologous chondrocyte implantation
- ☐ Osteochondral autograft plugs
- ☐ Osteochondral allograft
- ☐ Loose body removal
- ☐ Other

85.Which imaging modalities do you usually perform during follow-up after operative treatment for JOCD of the ankle? (multiple answers may be chosen)

- ☐ X-ray
- ☐ Ultrasound
- ☐ CT
- ☐ CT-arthrography
- ☐ SPECT
- ☐ MRI
- ☐ MR-arthrography
- ☐ None/only when indicated
- ☐ Other

86.Over what time period do you schedule follow-up visits after operative treatment of JOCD of the ankle?

- o 1 year
- o 2 years
- o 3 years
- o Until the end of growth
- o Other

87.Do you record any patient reported outcome measures (PROMs) after treatment for JOCD of the ankle? (multiple answers may be chosen)

- ☐ Yes, the AOFAS score
- ☐ Yes, the FAAM score

☐ Yes, the FAOS score
☐ Yes, the KAFS score
☐ No
☐ Other

Appendix 2: Case answers

Elbow – case 1

21. What is your preferred treatment for the following patient? (a combination of different techniques may be chosen that you would apply together)

    A boy, skeletal age 13, plays baseball two times a week and has pain in his right elbow (dominant side). Newly diagnosed with a primary OCD of 12mm of his capitellum. The lesion is radiologically unstable (articular cartilage is breached and there is fluid behind the subchondral bone on MRI) but not displaced.

More Details

Immobilization through bracing	0
Immobilization through casting	2
Non-steroidal anti-inflammator...	2
Injection therapy through cortic...	0
Injection therapy through platel...	0
Activity restriction/sports restric...	4
Internal fixation with non-absor...	1
Internal fixation with bioabsorb...	3
Bone grafting	0
Antegrade drilling	0
Retrograde drilling	0
Debridement	2
Microfracturing	2
Autologous chondrocyte implan...	0
Osteochondral autograft plugs	0
Osteochondral allograft	0
Loose body removal	1
Other	0

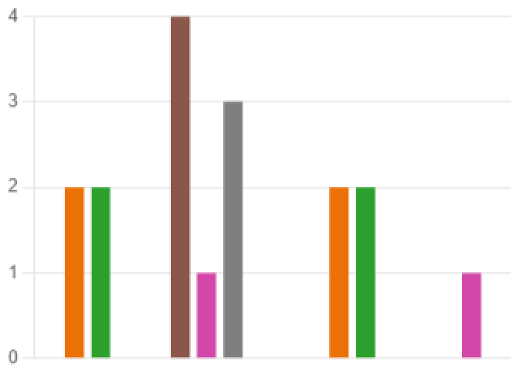

Elbow – case 2

22. What is your preferred treatment for the following patient? (a combination of different techniques may be chosen that you would apply together)

    A girl, skeletal age 11, does gymnastics three times a week and has pain in her right elbow (dominant side). Diagnosed with a primary OCD of 12mm of her capitellum six months ago. Sports restriction did not help. The lesion is radiologically stable (articular cartilage is breached and there is a low signal rim behind the fragment on MRI). There are no relevant changes on radiography, when comparing with the first images of six months ago.

More Details

Activity restriction/sports restric...	4
Immobilization through bracing	1
Immobilization through casting	1
Injection therapy through cortic...	0
Injection therapy through platel...	1
Non-steroidal anti-inflammator...	1
Internal fixation with non-absor...	0
Internal fixation with bioabsorb...	0
Bone grafting	0
Antegrade drilling	0
Retrograde drilling	2
Debridement	3
Microfracturing	2
Autologous chondrocyte implan...	0
Osteochondral autograft plugs	0
Osteochondral allograft	0
Loose body removal	1
Other	0

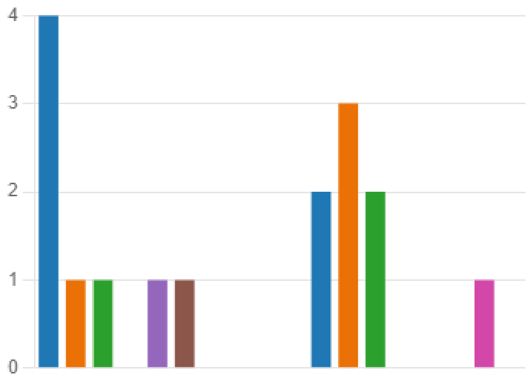

Knee – case 1

63. What is your preferred treatment for the following patient? (a combination of different techniques may be chosen that you would apply together)

A boy, skeletal age 13, plays football two times a week and has pain in his right knee. Newly diagnosed with a primary OCD lesion of 12mm of his medial femoral condyle on the right side. The lesion is radiologically unstable (articular cartilage is breached and there is fluid behind the subchondral bone on MRI) but not displaced.

More Details

Immobilization through casting	0
Immobilization through bracing	4
Immobilization through splinting	0
Limited weight bearing	12
Activity restriction	16
Injection therapy through cortic...	0
Injection therapy through platel...	2
Antegrade drilling	2
Transarticular drilling	3
Retroarticular drilling	1
Notch drilling	0
Internal fixation with non-absor...	11
Internal fixation with bioabsorba...	17
Bone grafting	6
Debridement	8
Microfracturing	3
Autologous chondrocyte implan...	1
Osteochondral autograft plugs	2
Osteochondral allograft	0
Loose body removal	2
Other	0

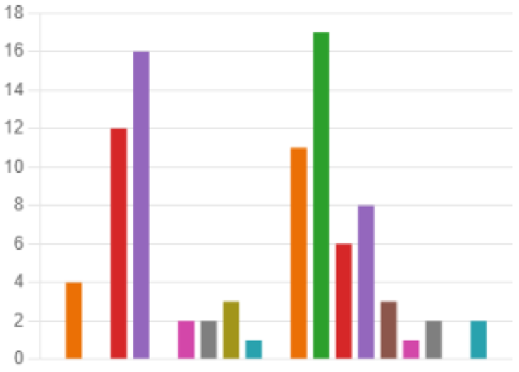

Knee – case 2

64. What is your preferred treatment for the following patient? (a combination of different techniques may be chosen that you would apply together)

    A girl, skeletal age 11, plays hockey three times a week and has pain in her right knee. Diagnosed with a primary OCD lesion of 12mm of her right medial femoral condyle six months ago. Sports restriction did not help. The lesion is radiologically stable (articular cartilage is breached and there is a low signal rim behind the fragment on MRI). There are no relevant changes on radiography, when comparing with the first images of six months ago.

More Details

Immobilization through casting	0
Immobilization through bracing	2
Immobilization through splinting	0
Limited weight bearing	14
Activity restriction	17
Injection therapy through cortic...	0
Injection therapy through platel...	4
Antegrade drilling	6
Transarticular drilling	4
Retroarticular drilling	9
Notch drilling	0
Internal fixation with non-absor...	2
Internal fixation with bioabsorba...	9
Bone grafting	1
Debridement	5
Microfracturing	4
Autologous chondrocyte implan...	0
Osteochondral autograft plugs	1
Osteochondral allograft	0
Loose body removal	3
Other	3

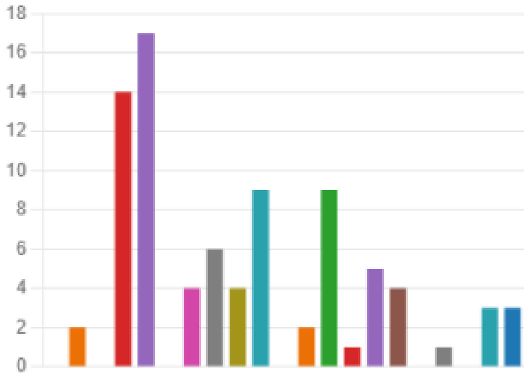

Ankle – case 1

83. What is your preferred treatment for the following patient? (a combination of different techniques may be chosen that you would apply together)

    A boy, skeletal age 13, plays football two times a week and has pain in his right ankle. Newly diagnosed with a primary OCD lesion of 12mm of the medial talar dome on the right side. The lesion is radiologically unstable (articular cartilage is breached and there is fluid behind the subchondral bone on MRI) but not displaced.

More Details

Immobilization through casting	2
Immobilization through bracing	3
Immobilization through splinting	1
Limited weight bearing	8
Activity restriction	10
Injection therapy through cortic...	0
Injection therapy through platel...	0
Internal fixation with non-absor...	1
Internal fixation with bioabsorba...	2
Drilling	2
Bone grafting	1
Debridement	3
Microfracturing	3
Autologous chondrocyte implan...	0
Osteochondral autograft plugs	0
Osteochondral allograft	0
Loose body removal	1
Other	1

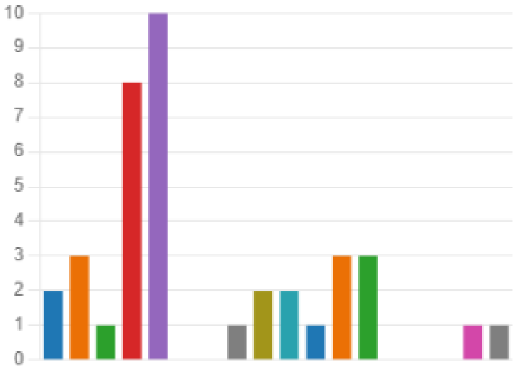

Ankle – case 2

84. What is your preferred treatment for the following patient? (a combination of different techniques may be chosen that you would apply together)

    A girl, skeletal age 11, plays hockey three times a week and has pain in her right ankle. Diagnosed with a primary OCD lesion of 12mm of the medial talar dome six months ago. Sports restriction did not help. The lesion is radiologically stable (articular cartilage is breached and there is a low signal rim behind the fragment on MRI). There are no relevant changes on radiography, when comparing with the first images of six months ago.

More Details

Immobilization through casting	1
Immobilization through bracing	2
Immobilization through splinting	0
Limited weight bearing	3
Activity restriction	5
Injection therapy through cortic...	0
Injection therapy through platel...	1
Internal fixation with non-absor...	2
Internal fixation with bioabsorba...	4
Drilling	3
Bone grafting	1
Debridement	1
Microfracturing	3
Autologous chondrocyte implan...	0
Osteochondral autograft plugs	0
Osteochondral allograft	0
Loose body removal	3
Other	1

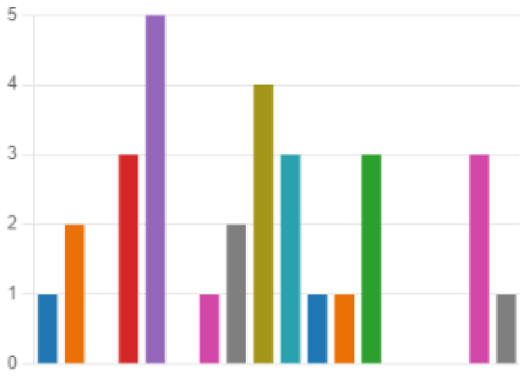

Supplement: Supplementary file 1 — Supplementary file1 (PDF 554 KB) [file 167_2023_7563_MOESM1_ESM.pdf]
